# Hypoglycaemia in adrenal insufficiency

**DOI:** 10.3389/fendo.2023.1198519

**Published:** 2023-11-20

**Authors:** Shien Chen Lee, Elizabeth S. Baranowski, Rajesh Sakremath, Vrinda Saraff, Zainaba Mohamed

**Affiliations:** ^1^ Department of Paediatrics, Princess Royal Hospital, Telford, United Kingdom; ^2^ Department of Paediatric Endocrinology, Birmingham Women’s and Children’s Hospital, Birmingham, United Kingdom; ^3^ Centre for Endocrinology, Diabetes and Metabolism, University of Birmingham, Birmingham, United Kingdom

**Keywords:** hypoglycaemia, adrenal insufficiency, hypoadrenalism, cortisol, glucocorticoid

## Abstract

Adrenal insufficiency encompasses a group of congenital and acquired disorders that lead to inadequate steroid production by the adrenal glands, mainly glucocorticoids, mineralocorticoids and androgens. These may be associated with other hormone deficiencies. Adrenal insufficiency may be primary, affecting the adrenal gland’s ability to produce cortisol directly; secondary, affecting the pituitary gland’s ability to produce adrenocorticotrophic hormone (ACTH); or tertiary, affecting corticotrophin-releasing hormone (CRH) production at the level of the hypothalamus. Congenital causes of adrenal insufficiency include the subtypes of Congenital Adrenal Hyperplasia, Adrenal Hypoplasia, genetic causes of Isolated ACTH deficiency or Combined Pituitary Hormone Deficiencies, usually caused by mutations in essential transcription factors. The most commonly inherited primary cause of adrenal insufficiency is Congenital Adrenal Hyperplasia due to 21-hydroxylase deficiency; with the classical form affecting 1 in 10,000 to 15,000 cases per year. Acquired causes of adrenal insufficiency can be subtyped into autoimmune (Addison’s Disease), traumatic (including haemorrhage or infarction), infective (e.g. Tuberculosis), infiltrative (e.g. neuroblastoma) and iatrogenic. Iatrogenic acquired causes include the use of prolonged exogenous steroids and post-surgical causes, such as the excision of a hypothalamic-pituitary tumour or adrenalectomy. Clinical features of adrenal insufficiency vary with age and with aetiology. They are often non-specific and may sometimes become apparent only in times of illness. Features range from those related to hypoglycaemia such as drowsiness, collapse, jitteriness, hypothermia and seizures. Features may also include signs of hypotension such as significant electrolyte imbalances and shock. Recognition of hypoglycaemia as a symptom of adrenal insufficiency is important to prevent treatable causes of sudden deaths. Cortisol has a key role in glucose homeostasis, particularly in the counter-regulatory mechanisms to prevent hypoglycaemia in times of biological stress. Affected neonates particularly appear susceptible to the compromise of these counter-regulatory mechanisms but it is recognised that affected older children and adults remain at risk of hypoglycaemia. In this review, we summarise the pathogenesis of hypoglycaemia in the context of adrenal insufficiency. We further explore the clinical features of hypoglycaemia based on different age groups and the burden of the disease, focusing on hypoglycaemic-related events in the various aetiologies of adrenal insufficiency. Finally, we sum up strategies from published literature for improved recognition and early prevention of hypoglycaemia in adrenal insufficiency, such as the use of continuous glucose monitoring or modifying glucocorticoid replacement.

## 1 Introduction

The adrenal glands are responsible for the production of 3 main steroids, mineralocorticoids (mainly aldosterone), glucocorticoids (mainly cortisol) and androgens. These are produced by the outer zona glomerulosa, middle zona fasciculata and inner zona reticularis of the adrenal cortex respectively. Cortisol and androgen productions are influenced by the hypothalamic-pituitary axis, whilst aldosterone is regulated by the renin-angiotensin system. Adrenal insufficiency (AI) occurs when there is primary adrenal failure or disruption to the hypothalamic-pituitary axis leading to inadequate steroid secretion. AI has different clinical presentations depending on the age and cause. Newborns with AI typically present with severe hypoglycaemia, seizures, failure to thrive, prolonged cholestatic jaundice, and in some cases coma. The lack of cortisol results in slow transport and maturation of bile acid synthesis, causing conjugated hyperbilirubinemia with raised liver enzymes, which usually presents at a median age of 13 days of life (1). In children and young adults with AI, they may have hypoglycaemia, weakness, fatigue, gastrointestinal symptoms, headaches, muscle and joint pains (1, 2). Symptoms may sometimes be non-specific such as postural hypotension, syncope, arthralgia, anorexia and mental health issues, as described in a case report of undiagnosed Addison’s disease who presented to the hospital in a collapsed state (3). Individuals with secondary adrenal insufficiency (SAI) due to abnormalities in the pituitary gland and hypothalamus may exhibit symptoms involving other hormone deficiencies or have coinciding midline defects. The risk of hypoglycaemia is increased if they have accompanying growth hormone (GH) deficiency, due to the counterregulatory role of GH in hypoglycaemia which is discussed later in this paper.

Adrenal crisis is a life-threatening complication of AI due to the body’s inability to respond to physiological stress. It is more common in PAI (primary adrenal insufficiency) compared to SAI (4, 5). Symptoms include hypotension, dehydration, vomiting, abdominal pain, and in the most serious cases, they may present with shock, coma and death (6). Unexplained sudden deaths in neonates and children should always raise the suspicion of an adrenal crisis. Data looking at hypoglycaemia-related deaths in patients with PAI is limited, however, there are published studies examining the effect of hypoglycaemia on the mortality rates of patients with SAI who are on growth hormones (7–9). In a large cohort study of patients who received growth hormone in the United States, 24.5% (106 out of the 433) deaths recorded were found to be sudden and unexpected. 74% of these unexpected deaths were associated with multiple pituitary hormone deficiencies and hypoglycaemia was highlighted in 31% of these 106 deaths. In addition, more than half of the unexpected deaths were thought to have undiagnosed secondary adrenal insufficiency (9).

Individuals with AI are at a higher risk of a hypoglycaemic event, especially when unwell or in an adrenal crisis. Hypoglycaemia can have serious consequences if left unrecognised and untreated. We aim to critically assess the burden of hypoglycaemia in the population of adrenal insufficiency and investigate ways to reduce morbidity and mortality in children with AI. We performed a literature search in Pubmed using the terms ‘primary adrenal insufficiency’, ‘secondary adrenal insufficiency’, ‘cortisol deficiency’, ‘hypoadrenalism’, ‘adrenal crises’, ‘hypoglycaemia’ and ‘low blood sugars’. Due to lack of reliable available evidence, we also included specific conditions known to cause AI for a more comprehensive review- mainly congenital adrenal hyperplasia (CAH), congenital adrenal hypoplasia, panhypopituitarism and Addison’s disease. All papers related to the search terms above were included in this review. In this manuscript, we have explored the counterregulatory mechanism of hypoglycaemia, reasons for individuals with AI to be more susceptible to hypoglycaemia, their clinical presentation and potential complications, current management and future developments.

## 2 Counterregulatory mechanism of hypoglycaemia

Under normal circumstances, when blood glucose falls below the physiological range, insulin production decreases and counterregulatory hormones are released to maintain glucose supply for vital bodily functions. The initial counterregulatory hormones released include glucagon and epinephrine. Both glucagon and epinephrine act by stimulating hepatic glycogenolysis and promoting gluconeogenesis. Epinephrine also limits glucose clearance by insulin-sensitive tissues (10).

Other counterregulatory hormones such as growth hormone and cortisol are secreted as blood glucose continues to fall. Both these hormones combat hypoglycaemia over a longer time frame. The role of growth hormone and cortisol in regulating the severity of hypoglycaemia during sustained intravenous insulin infusion was demonstrated in two studies by De Feo et. al (11, 12). Growth hormone and cortisol induce lipolysis in fat tissues and encourage hepatic ketogenesis and gluconeogenesis (13). Lipolysis causes an increase in free fatty acids which affects insulin signalling pathways (14). Cortisol also reduces insulin secretion from the pancreas (15). In glucose homeostasis, the rise of glucose leads to an increase in insulin production. Growth hormone also stimulates the production of insulin-like growth factor 1 (IGF-1) from the liver, provided that there is adequate nutrition and elevated portal insulin levels (16). Normoglycaemia is achieved as glucose is absorbed back into peripheral cells.

## 3 Hypoglycaemia in adrenal insufficiency

### 3.1 Aetiology

In general, children and young infants tend to be more vulnerable to the effects of hypoglycaemia. Neonatal hypoglycaemia can affect neurodevelopmental outcomes as neonates have higher brain glucose requirements, coupled with an immature pathway to respond to low blood sugars. Children have higher energy demand for growth and reduced glycogen supply compared to adults (17). Steady control of blood glucose is vital for neurocognitive development in children. A meta-analysis of children with Type 1 diabetes showed lower capabilities in memory and learning in those who had recurrent severe hypoglycaemic episodes compared to those who did not (18).

A few theories have been proposed to explain why individuals with AI are more susceptible to hypoglycaemia. Excess cortisol has been linked with an increase in insulin resistance (19, 20), possibly due to a rise in circulating fatty acids from cortisol-induced lipolysis (21, 22). Lack of cortisol has been shown to increase insulin sensitivity (23), which will enhance peripheral tissue’s uptake of glucose, thereby increasing the risk of a hypoglycaemia event. In addition, cortisol deficiency causes a reduction in endogenous glucose production and an increase in glucose oxidation, which can lead to hypoglycaemia (23). An impaired physiological rise in blood glucose levels during exercise was also seen in patients with classical CAH, even when they have good compliance with their glucocorticoid treatment. Doubling the hydrocortisone dose did not affect the exercise glycaemic levels in these cases (24).

Normal glucocorticoid secretion of the adrenal cortex is essential for optimal development and functioning of the adrenal medulla, which is responsible for the production of the majority of epinephrine hormone (25). Patients with CAH were found to have reduced epinephrine stores which may affect their counter-regulatory mechanism towards hypoglycaemia (26). A separate study has shown poorly developed adrenomedullary structures and fewer secretory vesicles in the chromaffin cells of patients with congenital adrenal hyperplasia (CAH) (27). Patients with AI are also more sensitive to hypoglycaemia due to the lack of epinephrine response during a hypoglycaemic event (28). Blunted epinephrine response was seen in all patients with pituitary adenoma during insulin-induced hypoglycaemia, but the impairment is worst in those patients with SAI (29).

Hypoglycaemia risk in children with SAI is dependent on the cause and presence of associated hormone deficiencies. In a case series, 3 children with a background of asthma who were on a high dose of inhaled corticosteroids, were found to have SAI after presenting with hypoglycaemia, accompanied by vomiting, drowsiness and tiredness. They suggested that adrenal suppression is influenced by patient susceptibility, dose and duration of the inhaled corticosteroids (30). Low cortisol levels during controlled hypoglycaemia in neonates with hyperinsulinism were reported in a clinical trial. This study demonstrated that hypocortisolaemia was due to inappropriately low ACTH levels, indicating that neonates with hyperinsulinism may have blunted ACTH response from the hypothalamic-pituitary axis during a hypoglycaemia event (31).

### 3.2 Clinical presentation and complications of hypoglycaemia in AI

Patients with AI are always at risk of an adrenal crisis. The incidence of adrenal crises in individuals with primary or secondary AI is approximately 5-10 per 100 patient-years (4, 5, 32). However, this incidence is higher in PAI (33). Reisch et al. reported more than 70% of these events occurred in the first 10 years of life in children with 21-hydroxylase deficiency (34). In a study following children with CAH up to 6 years of age, the number of adrenal crises is roughly 6.5 per 100 patient-years (35).

The frequency of hypoglycaemia during adrenal crises is not widely studied. In the adult population with AI, hypoglycaemia is less common. A study in Japan diagnosed adrenal insufficiency in 32 out of 528 patients (6%) after presenting to the emergency department with hypoglycaemia. Their symptoms include tremors, palpitations, sweating, hunger, paresthesia, dizziness, weakness, and confusion (36). Adult patients may also exhibit mild symptoms such as early morning headaches due to unrecognised nocturnal hypoglycaemia (37).

In children, a study conducted in France reported 30 out of 165 (18%) children and infants with PAI experienced hypoglycaemic episodes. Those who experienced hypoglycaemia had a significantly lower basal cortisol level. Additionally, all of these hypoglycaemic episodes were associated with prior fasting or poor oral intake due to vomiting or viral illness (38). In a study looking at emergency hospital admissions of children with CAH, 24 admissions (9%) were due to hypoglycaemia and a third of these presented with hypoglycaemic seizures (39). Pinto et al. reported about 8% of complications in CAH children were due to hypoglycaemia and these occurred between the ages of 1 and 3 years old (40). Besides that, a small study monitoring acute illness in children with CAH documented 3 out of 8 occasions of illness were associated with hypoglycaemia confirmed by capillary blood glucose monitoring. They all had lethargy as a common symptom (41).

In a cohort study following children with CAH up to their 6th year of life, hypoglycaemia episodes were recorded in 16 out of 102 children (15%). There were a total of 23 hypoglycaemic episodes and 7 of these episodes were associated with salt loss. Some of these children had more than one hypoglycaemic episode, suggesting varying individuals’ predispositions to hypoglycaemia. Loss of consciousness was seen in 13 out of the 16 hypoglycaemic episodes, and 5 children had hypoglycaemic seizures (35). In one study, 8 out of 63 (12%) children with Addison’s disease in a rural and urban Pakistan province presented with hypoglycaemia during an adrenal crisis (42).

One published cohort study in the UK from 1987 to 2017 showed a significant rise in mortality rates in patients with AI compared to matched healthy controls, and the risk is significantly higher in patients with PAI compared to SAI. Adrenal crisis was found to be an associated cause of death in 10% of those with adrenal insufficiency, and this was mainly due to a compromise in the circulatory system. Although it was difficult to ascertain if hypoglycaemia partly contributed to the adrenal crisis-associated deaths, it was identified that mortality rates in patients with AI and accompanying diabetes were higher than those with AI alone (43). Data from patients with CAH showed mortality rates in developed countries vary between 0 to 4% (44). One study calculated the standardized mortality ratio (SMR) to be 3 times higher in children with CAH, and the mortality was significantly higher in infancy and children up to 4 years old (45), especially in the salt-wasting phenotype (46, 47). An extensive 30-year study found a reduction in deaths from neonatal salt-wasting crises in the second half of their observation period, suggesting that there may be an improved understanding of the emergency management of CAH (46). It remains challenging to extrapolate if hypoglycaemia played a role in these salt-wasting crises. However, we found one other study that reported 2 deaths in young children with CAH from hypoglycaemic seizures (40).

### 3.3 Treatment and prevention

AI treatment aims to mimic the circadian rhythm, and in neonates, this is not fully established until about 3 months of age (48). The basal secretion of cortisol in children and young adults is known to be between 5-6mg/m2/day (1). Due to gastric acids and hepatic first-pass effect, the recommended glucocorticoid treatment in children with primary AI is slightly higher, ranging between 6-12mg/m2/day (1, 49). Children with secondary AI may require a lower daily replacement dose (6-8mg/m2/day) (49). An optimal balance of glucocorticoid regimen is vital as patients with AI will require lifelong steroid replacement. Overreplacement of glucocorticoids has been associated with increased mortality (50), and under-replacement may lead to adrenal crises and hypoglycaemia. One prospective 2-year study has closely monitored and titrated the doses of hydrocortisone based on salivary 17-OHP levels in children with CAH up to 8 years of age and the outcome was a lower median daily hydrocortisone dose with no observed adrenal crises (51). This stresses the importance of regularly monitoring all patients with AI on steroid replacement.

Hydrocortisone is the preferred glucocorticoid and it is usually given in 3 divided doses, with a bigger dose in the morning aiming to mimic the physiological circadian rhythm (1). However, this is challenging to achieve as the circadian cortisol level will usually start to rise in the early hours of the morning from 0200. Current conventional immediate-release hydrocortisone treatment is unable able to mimic this (49). Insulin sensitivity is also the highest between 0200 and 0400, causing patients with AI to be at risk of nocturnal hypoglycaemia (52).

**Figure 1:**
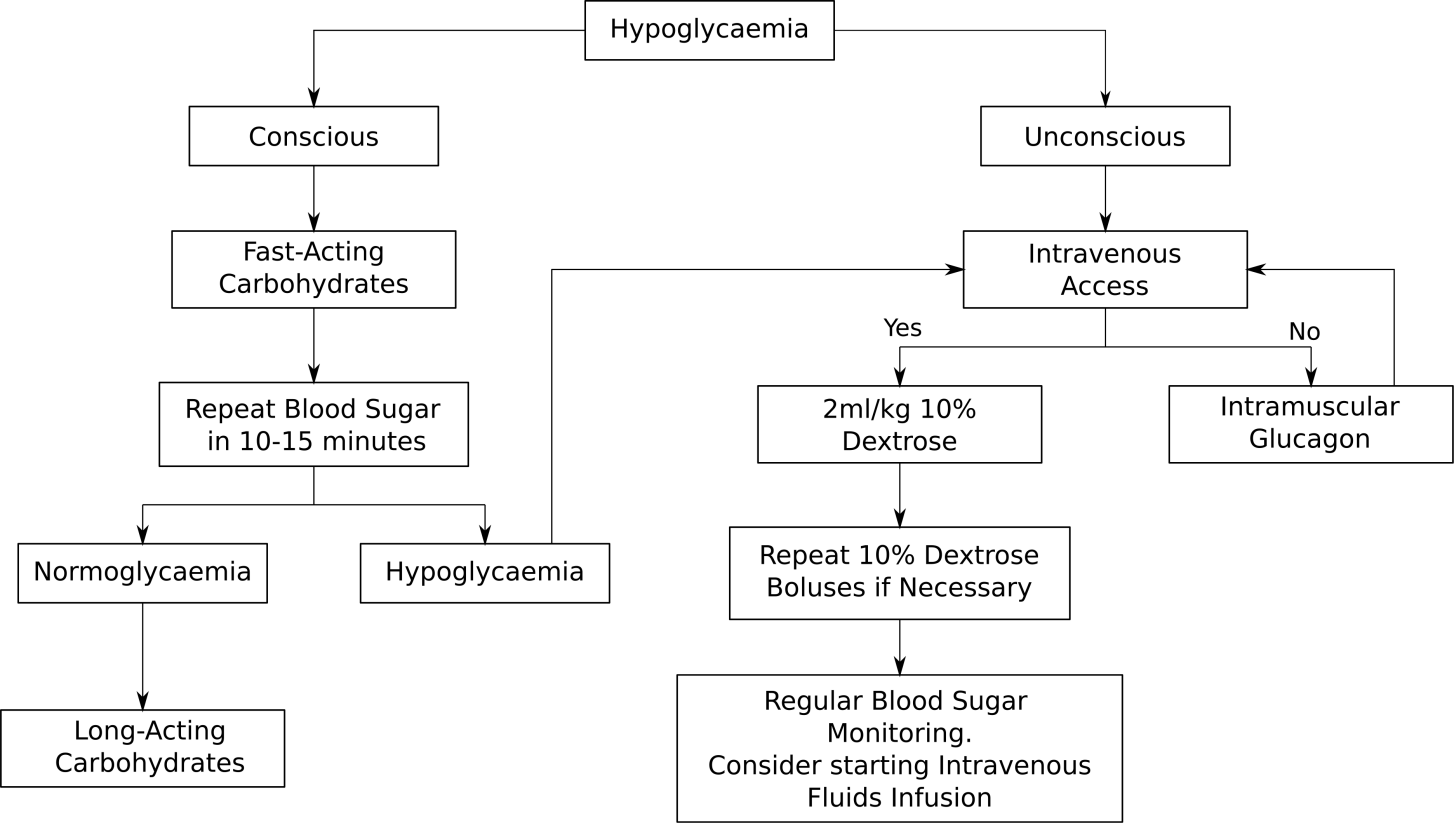
Emergency treatment of hypoglycaemia.

During an adrenal crisis, emergency hydrocortisone needs to be given immediately and this can be done via intramuscular injection (IM) or intravenously (IV). The doses of emergency hydrocortisone treatment are summarised in the above **Table 1** in keeping with the latest British Society for Paediatric Endocrinology and Diabetes (BSPED) guideline. If the child is hypoglycaemic but conscious, fast-acting carbohydrates such as sugary juice should be given, followed by long-acting carbohydrates such as biscuits. If the child is unconscious, a 2ml/kg fluid bolus of 10% dextrose should be given and the blood sugar should be repeated in 10 to 15 minutes. Repeat dextrose fluid boluses may be given if necessary. Administration of intramuscular glucagon could be considered if healthcare professionals are unable to obtain intravenous access. The emergency management of hypoglycaemia is outlined in **Figure 1** below. If a child with known AI is found to be hypoglycaemic and unconscious in the community, it is recommended that both intramuscular hydrocortisone and glucagon injections are administered immediately prior to arrival at the hospital.

**Table 1:** Emergency hydrocortisone doses as per BSPED 2023 guidelines.

Age of Child	Community	Hospital
Neonates (*<* 28 days)	25mg IM dose	4mg/kg IV initially 4−6 hourly Once stable: 2mg/kg IV 4−6 hourly
Children (*<* 1 year) Children (1−5 years) Children (*>* 6 years)	25mg IM dose 50mg IM dose 100mg IM dose	2mg/kg (max 100mg) IV initially 4−6 hourly Once stable: 1mg/kg (max 50mg) IV 4−6 hourly

The mainstay of early recognition and prevention of hypoglycaemia in adrenal insufficiency has been parental education. Children with AI are prone to hypoglycaemic events if they are not compliant with their steroid replacement (53). It is also important to educate parents/carers and older children with adrenal insufficiency on hydrocortisone stress dosing during an illness or injury (5). A National Patient Safety Alert has been issued in the UK for an emergency steroid card for all patients with AI, detailing clear instructions on what to do in an event of a suspected adrenal crisis. Despite these measures, a review study has found that parental education alone is not sufficient to reduce adverse events from adrenal crises (5). A recent qualitative study of parents with young children with AI demonstrated that most of them followed the hydrocortisone oral stress dosing instructions prior to hospital admission, but only 2 out of 16 parents gave parenteral hydrocortisone at home, and the remaining patients received this from either the paramedics or emergency department staff (54). Psychological barriers to giving intramuscular hydrocortisone still remain a challenge for most carers and patients with AI. The hope for the future is an alternate form of parenteral hydrocortisone such as subcutaneous hydrocortisone injection becoming available for use in children with AI (55).

### 3.4 Future directions

Recent years have seen the development of modified-release hydrocortisone (MR-HC). Both the once-daily (56) and twice-daily (57) modified-released versions have been shown to be a closer mimic of the physiological circadian rhythm, compared to the conventional multiple-dose regimen. The modified-release preparation has a dual-release mechanism; an outer coating for an immediate release of hydrocortisone, and an inner core for a more sustained slower release. Studies have reported a significant reduction in body mass index, improved lipid profile, better glycaemic control and improved quality of life in those who are in the MR-HC group (58, 59). In a recent clinical trial comparing immediate-release and MR-HC in patients with CAH, both groups were able to achieve better 24-hour 17-OHP levels at 24 weeks compared to baseline, thus failing to prove their primary outcome. However, an extension of this clinical trial showed an overall improvement in disease control in the MR-HC group due to lower 17-OHP and androstenedione levels at multiple points throughout the day, with a significant reduction in 17-OHP variability. Dose reduction was also achievable in the MR-HC group whilst maintaining good disease control (60). In the United Kingdom, MR-HC has recently been approved for use in children and adolescents from 12 years of age with CAH. It will be exciting to see if this new modified-release therapy can reduce the incidence of hypoglycaemia in children with AI, especially during the early hours of the morning when they are most susceptible.

Identification of children with AI who experience recurrent nocturnal hypoglycaemia is also important to reduce occurrences. Continuous blood sugar monitoring (CGM) is currently mostly used in children with Type 1 diabetes, and less commonly used in those with Type 2 diabetes who are on insulin. Several studies conducted using CGM in adults with AI have shown benefits as they provided useful information on blood sugar trends. One study detected unrecognised nocturnal hypoglycaemia in 5 out of 6 adults with central hypoadrenalism causing early morning headaches. Full resolution of symptoms was achieved when the timings and dosages of the patients’ hydrocortisone were altered based on the CGM readings (37). In a separate case study, a patient with Addison’s disease reported better sleep when the evening dose of hydrocortisone was delayed due to detected hypoglycaemia from CGM (52). CGM in children with combined congenital central adrenal insufficiency and growth hormone deficiency have identified severe asymptomatic nocturnal hypoglycaemia (*<* 2.7mmol/L) in 3 out of 11 children, with the period of hypoglycaemia ranging from 30 to 150 minutes. On further review, they found that the daily doses of hydrocortisone were significantly lower in the 3 children and resolution of hypoglycaemia was seen when the doses were increased (49).

So why is CGM not widely used in hypoglycaemia disorders apart from diabetes? In a recent review by Worth et al. on the use of CGM, they found that hypoglycaemia sensitivity reduces as the threshold for hypoglycaemia decreases (61). The lowest hypoglycaemia sensitivity was reported at 17% with a hypoglycaemia threshold of 2.6mmol/L (62), as opposed to the highest hypoglycaemia sensitivity of only 86% with a hypoglycaemia threshold of 3.9mmol/L (63). The accuracy of CGM during a hypoglycaemia event is also variable and dependent on a lot of factors such as insulin levels and rapid glucose changes (64). Nevertheless, advancements in technology and machine learning methods continue to improve and streamline hypoglycaemia predictive algorithms to improve accuracy. There is definitely a scope for the broader use of CGM, especially in children with recurrent hypoglycaemia. A recent large survey in the UK revealed a high demand for CGM in those patients with recurrent hypoglycaemia without diabetes and it was interesting to see how funding was obtained for some of these patients across the country. Parents and carers also described marked improvement in the quality of life with CGM use and a reduction in unplanned hospital admissions due to hypoglycaemia (65). In summary, the definitive role of CGM in the prevention of hypoglycaemia in children with AI is yet to be established and calls for larger multi-centre studies.

## 4 Conclusion

We have summarised the current literature on hypoglycaemia in AI and the significance of this problem as children are vulnerable to the effects of hypoglycaemia, especially young children with AI. We have discussed a few strategies to improve recognition of hypoglycaemia in individuals with AI including the use of CGM. However, the use of technology for blood sugar monitoring in children is currently only established in Type 1 diabetes. Future research is needed to utilise the advancement of technology in the management of AI and expand the role of CGM in children with AI. In the meanwhile, perhaps intermittent capillary or flash glucose monitoring in detecting those children with AI at risk of hypoglycaemia might be beneficial, with the hope that this will improve their neurodevelopmental outcome and reduce mortality rates from hypoglycaemia.

## Author contributions

ZM had the presented idea. EB developed and outlined the abstract. SL performed the literature search and wrote the article. All authors contributed to the final manuscript, read and approved the submitted version.
